# Supplementary figures and images for: The COMBAT-TB Workbench: Making Powerful Mycobacterium tuberculosis Bioinformatics Accessible
Source: mSphere. 2022 Feb 9;7(1):e00991-21. doi: 10.1128/msphere.00991-21 (PMC8827006; doi:10.1128/msphere.00991-21)

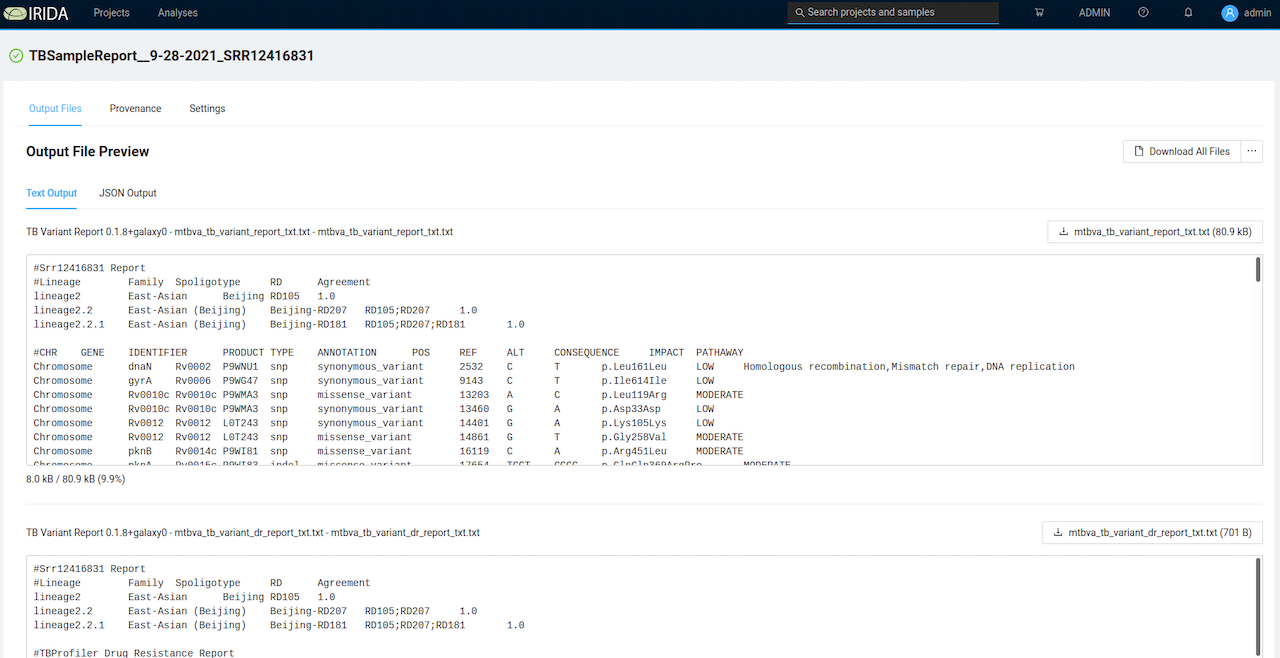

Supplement: FIG S1 [file msphere.00991-21-sf001.tif]

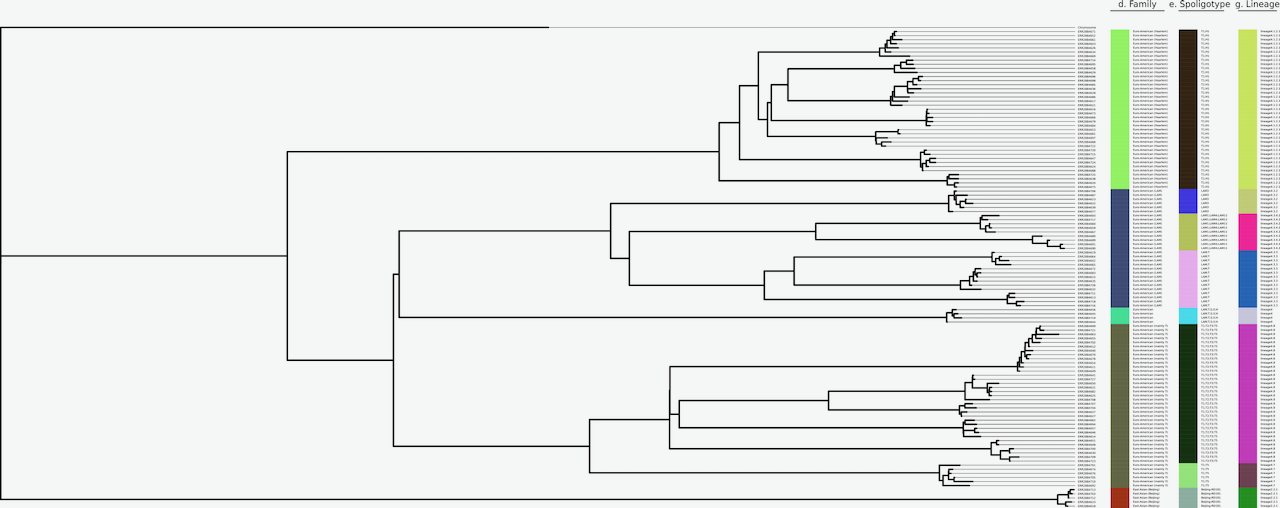

Supplement: FIG S2 [file msphere.00991-21-sf002.tif]
